# Study on the Preparation and Properties of Thermally Conductive Semi-Aromatic Heat-Resistant PA5T-CO-10T/ Hexagonal Boron Nitride Composites

**DOI:** 10.3390/polym17081031

**Published:** 2025-04-10

**Authors:** Bingxiao Liu, Yunzhen Zhu, Chen Yang, Liqun Ma, Fuchun Zhang, Mingzheng Hao, Zhongqiang Wang, Lizhen Bai, Jiale An, Dongqi Xiao

**Affiliations:** 1 Department of Materials Engineering, Taiyuan Institute of Technology, Taiyuan 030008, China; zhuyunzhen0335@163.com (Y.Z.); lqma0827@163.com (L.M.); haomz@tit.edu.cn (M.H.); bailizhen1234@163.com (L.B.); 18803479573@163.com (J.A.); 19135215294@163.com (D.X.); 2 Shanxi Center of Technology Innovation for Polyamide Materials, Taiyuan 030000, China; 19135127929@163.com; 3 Research and Development Center, Cathay Biotech Inc., Shanghai 201114, China; 4 Technology Center, Guangdong Sinoplast Advanced Material Co., Ltd., Dongguan 523879, China; wangzq@sinoplast.com.cn

**Keywords:** bio-based materials, copolymer, semi-aromatic polyamide, heat-resistant polymer

## Abstract

In this paper, we report a novel thermally conductive semi-aromatic heat-resistant PA5T-CO-10T/hexagonal boron nitride (PA5T-CO-10T/BN) composite, based on as-synthesized PA5T-CO-10T, which is a copolymer of poly (pentamethylene terephthalamide) (PA5T) and poly (decamethylene terephthalamide) (PA10T). We confirmed the structure of PA5T-CO-10T through a nuclear magnetic resonance carbon spectrometer (^13^C-NMR). The differential scanning calorimetry (DSC) and thermogravimetric analysis (TGA) results indicate that PA5T-CO-10T demonstrates a processing window (greater than 90 °C) which is suitable for melt processing and injection molding. Moreover, the PA5T-CO-10T composites with different BN contents were tested by scanning electron microscopy (SEM), a thermal conductivity meter, a rotational rheometer and X-ray diffraction (XRD). The results indicate that as the content of h-BN increases, the thermal conductivity of the PA5T-CO-10T/BN composites is significantly enhanced. When the mass of h-BN reaches 30 wt%, the thermal conductivity of the composite material is 2.5 times that of the original matrix resin. Simultaneously, there is a notable upward trend observed in the storage modulus, loss modulus, complex viscosity and orientation degree of h-BN. This is attributed to the high thermal conductivity and the high orientation degree of h-BN, which ensure the continuous enhancement of the material’s thermal conductivity. Additionally, the introduction of h-BN enhances the degree of connection between the material’s molecular chains. PA5T-CO-10T/BN possesses excellent heat resistance and thermal conductivity, presenting significant application prospects in the fields of electronics, electrical appliances and automobiles.

## 1. Introduction

Semi-aromatic polyamide, which combines the excellent processability of aliphatic polyamide with the superior heat resistance of aromatic polyamide (much higher than that of common polymers such as polylactic acid (PLA), polypropylene (PP) and polyethylene (PE)), is widely used in the mechanical field (e.g., in hydraulic cylinder pistons and structural components), the automotive field (e.g., in turbo air ducts and cylinder head covers) and the electronic and electrical fields (e.g., in LED reflective brackets and wire to board connectors) [1,2,3,4,5,6]. Currently, among the commercially applied semi-aromatic polyamides (copolymers of poly (hexanediamine terephthalamide) PA6T, poly (nonanediamine terephthalamide) PA9T and poly (decanediamine terephthalamide) PA10T), PA6T boasts superior heat resistance and a lower cost, thus enjoying a larger market share [4,7,8]. However, the development and further application of the petroleum-based product PA6T are constrained by the severe environmental issues arising from the rapid depletion of fossil fuels [9,10,11].

1,5-pentanediamine and 1,6-hexanediamine share similar structures, and it can be synthesized from lysine through the enzymatic biotransformation in cells or prepared through the biological fermentation of corn or straw [12]. Moreover, Cathay Biotech Inc. (Taiyuan, China) has already achieved the industrialization of 1,5-pentanediamine. Thus, we prepared bio-based semi-aromatic PA5T using 1,5-pentanediamine and terephthalic acid as the main raw materials through a salt-forming + solid-state polycondensation method. Regrettably, we found that its thermal decomposition initiation temperature is close to its melting temperature, making it impossible to perform the melting processing and injection molding. Consequently, we introduced the bio-based long carbon chain 10T segment into the backbone of the 5T molecule and obtained the PA5T-CO-10T copolymer, which possesses a relatively wide processing window.

Semi-aromatic heat-resistant polyamide is typically used at high temperatures. An outstanding thermal conductivity can better ensure the efficient operation of electrical products at lower temperatures. However, the thermal conductivity of PA5T-CO-10T is relatively low (0.1784 W·m^−1^·K^−1^), which cannot meet the thermal management requirements of electronic components [13]. Generally, melt blending is one of the most common and effective methods to improve polymer properties [14,15].

Hexagonal boron nitride (h-BN) is composed of equal amounts of boron (B) and nitrogen (N) atoms. It has a crystal structure similar to that of graphite, exhibiting an atomic crystal form and a dense structure [16,17]. It primarily conducts heat through phonons, boasting a high thermal conductivity [18,19]. In addition, its thermal expansion coefficient is the smallest among ceramics, and it also possesses excellent high-temperature insulation properties, making it a good high-insulation and high-thermal conductivity filler [20,21,22]. The addition of h-BN can effectively enhance the thermal conductivity of the matrix resin. Its good interfacial compatibility with polymers, as well as its excellent dispersibility and moderate orientation within the matrix resin, are the guarantees for achieving good thermal conductivity and thermal management capabilities in the composite material. Interestingly, the majority of current reports on thermally conductive polymer-based materials are concentrated in the area of general-purpose engineering plastics, whereas there is a scarcity of reports on bio-based high-temperature-resistant thermally conductive materials.

Based on the aforementioned background, this study aimed to prepare PA5T-CO-10T/BN composites that exhibit excellent heat resistance, processability and thermal conductivity, which was prepared by blending the as-synthesized PA5T-CO-10T with surface-treated h-BN. Scanning electron microscopy (SEM) was utilized to observe the morphological structures of PA5T-CO-10T/BN. The influence of the h-BN content on the thermal conductivity, storage modulus, loss modulus, complex viscosity and crystallization properties of the composite materials was also investigated. This research will provide new insights into the high-value-added applications of PA5T-CO-10T and offer new material options for fields such as electronics, electrical appliances and automobiles.

## 2. Materials and Methods

### 2.1. Materials

The 1,5-pentanediamine was obtained from Cathay Biotech Inc. (Shanghai, China). The 1,10-diaminodecane was purchased from Wuxi Yinda Nylons Co., Ltd. Shanghai Ma Kelin Biochemical Technology Co. (Wuxi, China). provided the terephthalic acid, silane coupling agent (KH550) and antioxidant 1010. The h-BN, anhydrous ethanol and deuterated trifluoroacetic acid (the solvent for the nuclear magnetic resonance testing) were purchased from Shanghai Aladdin Bio-Chem Technology Co., Ltd. Shanghai, China. The deionized water was self-made.

### 2.2. The Synthesis of PA5T-CO-10T

Based on our previous research, and in order to further promote the application of PA5T-CO-10T as well as to meet the demand for preparing high-thermal-conductivity PA5T-CO-10T/BN composites with varying h-BN contents, we conducted a slightly larger batch synthesis of PA5T-CO-10T. The specific process was as follows [23]: We weighed 1,5-pentanediamine, 1,10-decanediamine and terephthalic acid according to the molar ratios of 1:0:1 and 0.5:0.5:1, respectively. We then placed the raw materials in a reaction vessel and added an appropriate amount of deionized water. We turned on the stirrer and gradually heated the reaction system to above 60 °C. We allowed the reaction to proceed at this temperature for 2 h. Subsequently, we placed the sample in a vacuum oven (DZF-6055 type, Shanghai Yiheng Scientific Instrument Co., Ltd., Shanghai, China) for drying to obtain the PA5T and PA5T-CO-10T salts.

The PA5T and PA5T-CO-10T salts were placed separately in a high-temperature and high-pressure polymerization reactor (XSF-10 type, Weihai Xingyu Chemical Machinery Co., Ltd., Weihai, China). We gradually increased the temperature of the reaction system to 220–230 °C. After the pressure stabilized, we maintained the temperature and pressure for a reaction of 1 h. The pressure of the reaction system should be gradually released to −0.09 MPa and the temperature raised to 260 °C. Under these temperature and pressure conditions, the reaction continues for 3–4 h, resulting in the production of PA5T and PA5T-CO-10T.

### 2.3. Surface Treatment of h-BN

Anhydrous ethanol, deionized water and silane coupling agent KH550 were mixed in a beaker at a mass ratio of 6:3:1, followed by the addition of an appropriate amount of h-BN. The mixed solution was subjected to ultrasonic stripping for 10 min using an ultrasonic cleaning machine (PS-40AL model, Dandong Tongda Instrument Co., Ltd., Dandong, China). Then, the mixture was placed in a three-neck flask and the stirrer was turned on and gradually heated to 80 °C then allowed to react for 2 h (To ensure the stability of the reaction system, a condensation reflux device needed to be installed). After the reaction was complete, a Buchner funnel was used for the suction filtration, and then the sample was placed into a vacuum drying oven at 100 °C for further drying and reaction for 10 h. Subsequently, the products were repeatedly cleaned using anhydrous ethanol to effectively remove impurities. After the cleaning, the samples were placed in a vacuum drying oven at 100 °C for drying, resulting in KH550-BN.

### 2.4. Preparation of PA5T-CO-10T/BN

KH550-BN, antioxidant 1010 and PA5T-CO-10T were weighed and mixed according to the mass ratio in Table 1 and then dried in a vacuum drying oven at 130 °C for 6 h. The mixture was melt blended using a micro twin-screw extruder (WLG10AG type, Shanghai Xinshuo Precision Machinery Co., Ltd., Shanghai, China) with the upper and lower cavity temperatures set at 305 °C and a rotational speed of 24 r/min, resulting in PA5T-CO-10T/BN composites with varying h-BN contents. Next, the PA5T-CO-10T/BN composites with different h-BN contents were injection molded using a micro injection molding machine (WZS10D type, Shanghai Xinshuo Precision Machinery Co., Ltd., Shanghai, China) with a barrel temperature of 305 °C and a mold temperature of 90 °C.

### 2.5. Characterization

#### 2.5.1. Nuclear Magnetic Resonance Carbon Spectrometer (^13^C-NMR) Test

The PA5T and PA5T-CO-10T samples were dissolved separately in a deuterated trifluoroacetic acid reagent, and then ^13^C-NMR testing was conducted using a nuclear magnetic resonance spectrometer (DPX-400 model, Bruker Company, Karlsruhe, Germany) to confirm the structure of PA5T and PA5T-CO-10T.

#### 2.5.2. Thermogravimetric Test

The TGA of PA5T and PA5T-CO-10T were performed using an STA449 (Netzsch company, Selb, Germany) under a nitrogen atmosphere. The sample was placed in the crucible and heated from 30 °C to 700 °C at a rate of 10 °C/min, and the thermogravimetric curves were recorded.

#### 2.5.3. Differential Scanning Calorimetry (DSC) Test

The DSC measurements of PA5T and PA5T-CO-10T were carried out on a Q20 (TA Instruments, New Castle, DE, USA) in a nitrogen atmosphere. We placed 3–5 mg of the PA5T or PA5T-CO-10T sample in crucibles, heated them at a rate of 40 °C/min until the samples reached a temperature above their melting point, and maintained this for 5 min to eliminate the thermal history. They were then cooled to 30 °C at a rate of 10 °C/min. After maintaining this temperature for 5 min, the temperature was raised again at a rate of 10 °C/min to the temperature above the polymer’s melting point, and the DSC curves were recorded.

#### 2.5.4. Fourier Transform Infrared Spectroscopy (FTIR) Test

h-BH and KH550-BN were separately mixed with potassium bromide, followed by grinding and tabletting. The FTIR (model S50, Thermo Nicolet, Thermo Fisher Scientific, Waltham, MA, USA) was used for testing in the transmissive mode, with a scanning range of 4000–500 cm^−1^.

#### 2.5.5. SEM Test

To further investigate the dispersion of h-BN in PA5T-CO-10T and the interfacial bonding between h-BN and PA5T-CO-10T, the PA5T-CO-10T/BN samples with different h-BN contents were first gold-coated. Subsequently, SEM (SU3500, Hitachi, Tokyo, Japan) was used to analyze the microstructure of the samples at a voltage of 15 kV under magnifications of 1000× and 5000×, respectively.

#### 2.5.6. Thermal Conductivity Test

The samples (round, with a diameter of 20 mm and a thickness of 2 mm) after injection molding were tested using a thermal conductivity meter (TPS 2500S model, HotDisk, Gothenburg, Sweden) at a test temperature of 25 °C. Based on the standard of ISO 22007 [24] and the transient plane source method, the thermal conductivities of the PA5T-CO-10T composites with different h-BN contents were calculated.

#### 2.5.7. Rotational Rheometer Test

The injection-molded discs (diameter 20 mm, thickness 2 mm) were placed on the rotor inside the cavity of the rotational rheometer (ARES-G2, TA, New Castle, DE, USA), and temperature scanning was performed on the PA5T-CO-10T/BN composites with different h-BN contents. The scanning range was 280–300 °C. The trends of the storage modulus, loss modulus and complex viscosity of the samples with different formulations were recorded, as they varied with the temperature.

#### 2.5.8. X-Ray Diffractometer (XRD) Test

To investigate the influence of the h-BN content on the crystallization properties of the polymers and understand the orientation of h-BN in the matrix resin, samples with different formulations were placed in test grooves, fixed and then characterized using XRD (SmartLab SE model, Rigaku, Japan) to record the WAXD diffraction patterns. The scanning range was 5–60 ° with a speed of 10 °/min.

## 3. Results and Discussion

### 3.1. ^13^C-NMR Analysis of PA5T and PA5T-CO-10T

**Figure 1:**
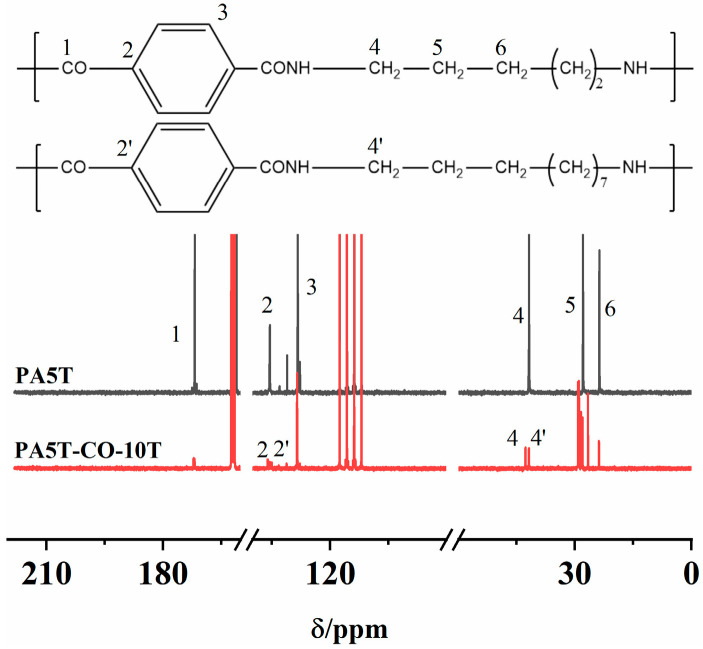
^13^C NMR spectra of PA5T and PA5T-CO-10T.

The ^13^C-NMR spectra of PA5T and PA5T-CO-10T are shown in Figure 1. The peak near 170.98 ppm could be attributed to the carbon atom on the carbonyl group (position 1). The chemical shift near 134.70 ppm is caused by the carbon atom on the benzene ring connected to the carbonyl group (position 2). The peak at approximately 127.53 ppm corresponds to the carbon atom at position 3 of the benzene ring. The peaks at 40.98 ppm (position 4) and within the range of 22.85–27.08 ppm (positions 5–6) originate from the proton signals of carbon atoms on the methylene group connected to the amide group N atom and the carbon atoms on the remaining methylene groups, respectively. Interestingly, it could be discovered that PA5T-CO-10T exhibits additional peaks at positions 2′ and 4′ compared to PA5T, which is attributed to the irregular copolymerization of PA5T and PA10T [25]. The aforementioned peak positions align with the theoretical peak positions of PA5T and PA5T-CO-10T, thereby verifying the chemical structures of PA5T and PA5T-CO-10T [26].

### 3.2. FTIR Analysis

**Figure 2:**
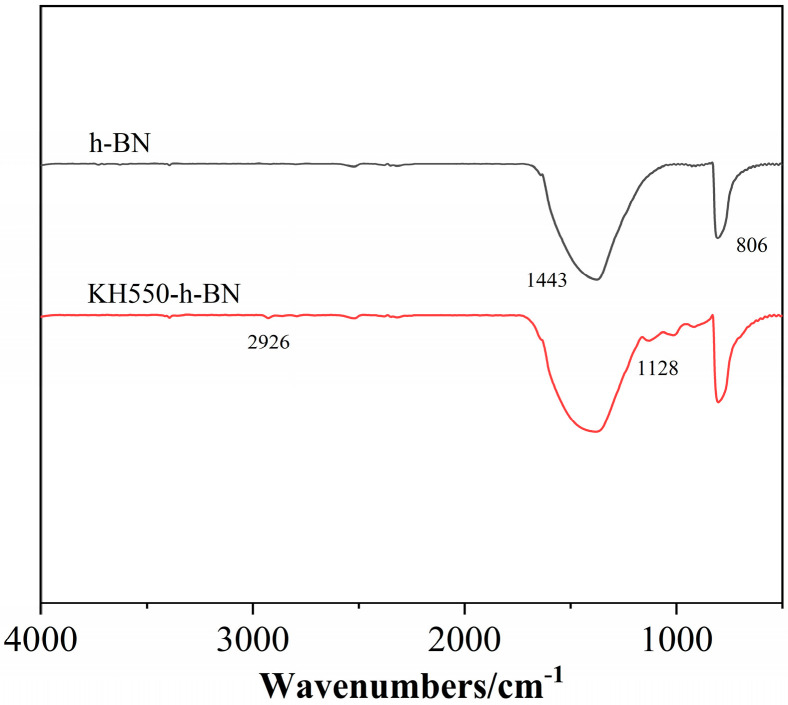
FTIR spectra of h-BN and KH-550-h-BN.

Figure 2 displays the infrared spectra of h-BH and h-BH treated with the silane coupling agent (KH550-BN) [27]. The characteristic peaks at 1443 cm^−1^ and 806 cm^−1^ correspond to the stretching vibration absorption peak and the bending vibration peak of BN, respectively [28]. As indicated in Figure 2, the spectrum of KH550-BN exhibits additional peaks at 1128 and 2926 cm^−1^ compared to that of h-BH, which correspond to the Si-O-Si asymmetric stretching vibration peak and the stretching vibration absorption peak of the methylene group on the silane coupling agent after binding with h-BH, respectively, indicating that KH550 was successfully grafted onto BN after hydrolysis [29].

### 3.3. SEM Analysis

**Figure 3:**
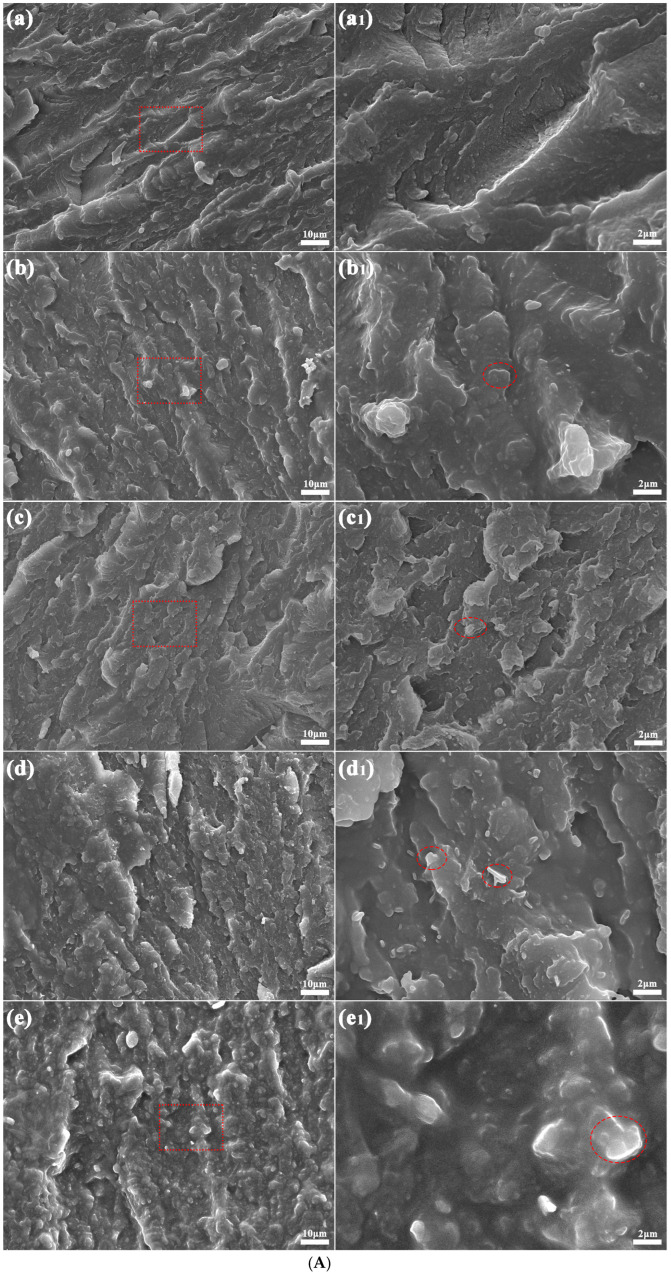

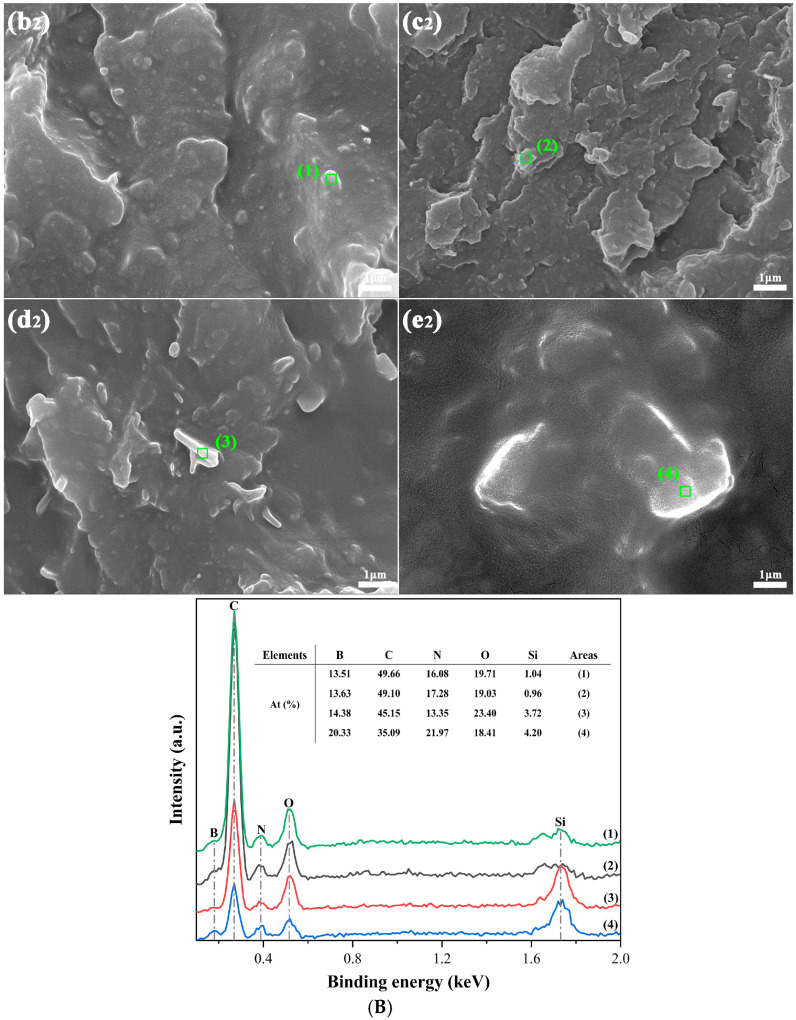
(**A**) SEM graphs of PA5T-CO-10T/BN composites with different h-BN contents. (**B**) EDS analysis of PA5T-CO-10T/BN composites with different h-BN contents (×10,000). (**a**,**a1**): pure PA5T-CO-10T; (**b**,**b1**,**b2**): PA5T-CO-10T/5% BN; (**c**,**c1**,**c2**): PA5T-CO-10T/10% BN; (**d**,**d1**,**d2**): PA5T-CO-10T/20% BN; (**e**,**e1**,**e2**): PA5T-CO-10T/30% BN.

Figure 3 depicts the microstructural morphologies of the PA5T-CO-10T/BN composites with varying h-BN contents. The imaging analysis was performed at two distinct magnifications: 1000× (left) and 5000× (right) (partial enlarged view of the area within the red dashed square frame in the left image). Compared to the pure PA5T-CO-10T, there are some lamellar areas with irregular geometry (as shown in the red dashed oval frames) in the fractured surface of the PA5T-CO-10T/BN composites, which are considered as two-dimensional h-BN additives. The EDS spectra and analysis results (shown in Figure 3B) show that the areas above contain a certain amount of B, C, N, O and Si elements, which belong to the modified hexagonal boron nitrides. Lamellar h-BN is uniformly dispersed within the composite material with almost no clusters (as shown in Figure 3A), which is attributed to the strong interfacial strength between h-BN and the resin matrix caused by the polyamide groups and hydrogen bonds. It can also be observed that as the content of h-BN increases, the spatial proportion occupied by boron nitride becomes larger, which also provides conditions for forming thermal conductive pathways in the polymer system [30,31]. Moreover, most of the h-BN particles exhibit in-plane orientation, which is due to the shear stress generated during the injection molding process, causing the h-BN particles to exhibit an oriented arrangement in the composite material. The orientation of h-BN makes it easier to form thermal conductive pathways, further ensuring the thermal conductivity of the composite material.

### 3.4. Thermal Performance Analysis

**Figure 4:**
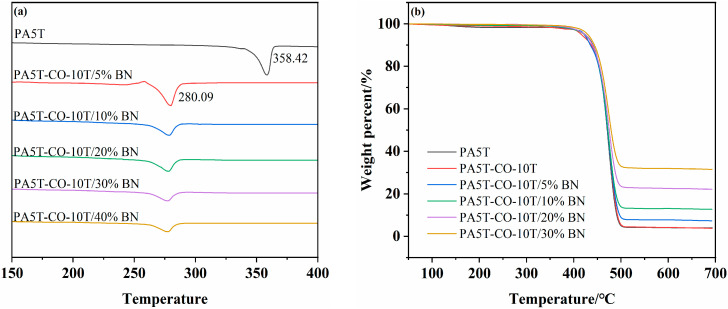
(**a**) DSC diagrams of PA5T and PA5T-CO-10T. (**b**) TGA (**b**) diagrams of PA5T and PA5T-CO-10T.

Figure 4a,b present the DSC and TG curves of PA5T, PA5T-CO-10T, and PA5T-CO-10T/BN, respectively. As shown in Figure 4a, the melting temperature of PA5T-CO-10T (280.09 °C) is significantly lower than that of PA5T (358.42 °C), which is attributed to the introduction of PA10T segments reducing the rigidity and regularity of the molecular chains. Meanwhile, it can be observed that the addition of h-BN does not significantly affect the melting temperature of the material. Notably, the melting temperatures of both PA5T-CO-10T and PA5T-CO-10T/BN are above 275 °C, fully meeting the industry’s requirements for heat-resistant materials [25,32]. As depicted in Figure 4b, the TG curves of PA5T and PA5T-CO-10T almost overlap, indicating that their initial thermal decomposition temperatures are nearly identical [5]. This similarity arises from their comparable chemical compositions, both belonging to the polyamide system, leading them to exhibit similar basic characteristics in terms of thermal stability. Compared with PA5T, both PA5T-CO-10T and PA5T-CO-10T/BN exhibit a wider processing window (greater than 90 °C; the initial thermal decomposition temperature minus the melting temperature).

### 3.5. Thermal Conductivity Analysis

**Figure 5:**
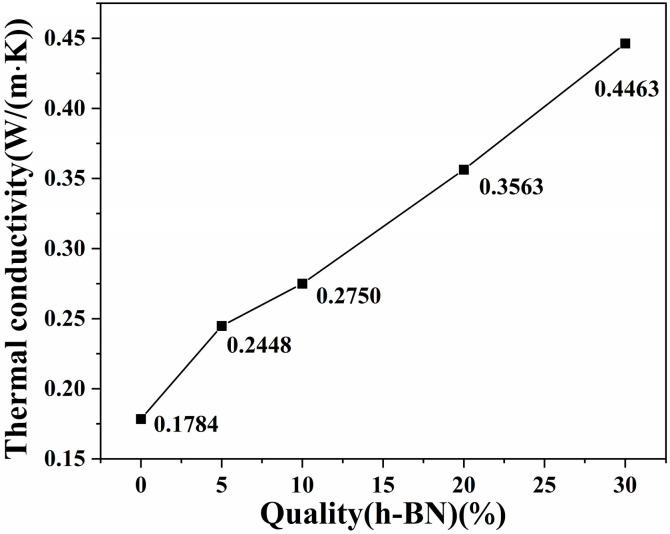
Effect of h-BN content on PA5T-CO-10T/BN composite.

Figure 5 illustrates the thermal conductivity of the PA5T-CO-10T/BN composites with varying h-BN contents. As indicated in Figure 5, the thermal conductivity of pure PA5T-CO-10T resin is relatively low, only reaching 0.1784 W·m^−1^·K^−1^. With the increase in the h-BN content, the thermal conductivity of the PA5T-CO-10T/BN composites generally exhibits a relatively rapid growth rate. Specifically, when the h-BN filling amount is 30 wt%, the thermal conductivity of the composite reaches 0.446 W·m^−1^·K^−1^, which is 2.5 times that of the pure PA5T-CO-10T resin, reaching the level reported in the relevant literature [33,34]. This is due to the fact that, according to the SEM analysis, h-BN demonstrates excellent dispersion and interfacial bonding within the matrix resin. Consequently, with the increase in the content of h-BN, which possesses good thermal conductivity, it becomes easier to form thermally conductive pathways among the matrix resins [35]. These factors collectively provide a guarantee for the continuous and effective enhancement of the thermal conductivity of PA5T-CO-10T.

### 3.6. Rotational Rheological Test Analysis

**Figure 6:**
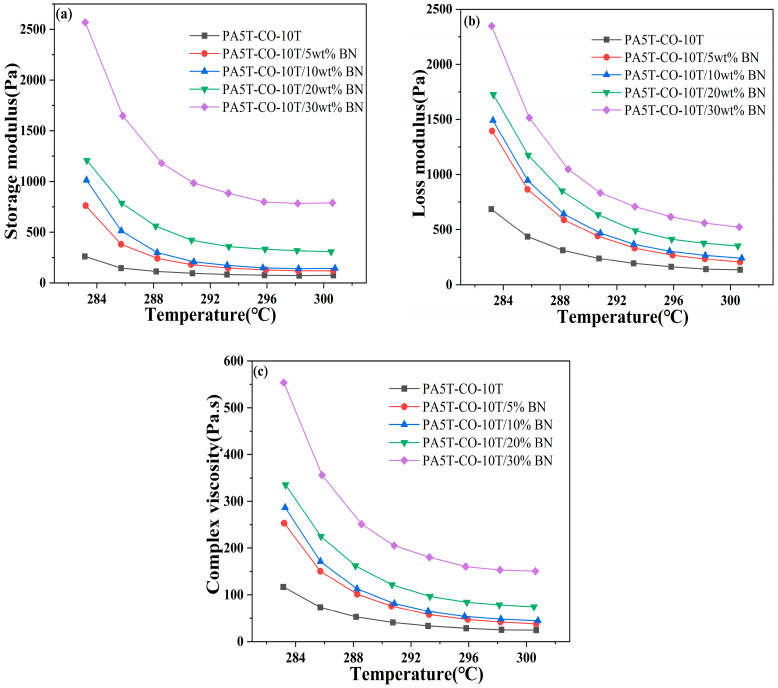
Analysis of storage modulus (**a**), loss modulus (**b**) and viscosity (**c**) of PA5T-CO-10T/BN composite material.

The trends of the storage modulus, loss modulus and complex viscosity of the PA5T-CO-10T/BN composites with varying h-BN contents as a function of temperature are shown in Figure 6a–c, respectively. As can be seen in Figure 6a, the storage modulus of the PA5T-CO-10T/BN composites with different h-BN contents decreases as the temperature increases, until it stabilizes at a certain value. This is due to the gradual melting of the matrix resin as the temperature rises, making the material more susceptible to deformation under stress, thus leading to a gradual decrease in its storage modulus [36]. When the resin is fully melted, the value of the storage modulus becomes relatively stable. It can also be observed from the figure that the storage modulus of the material exhibits a gradual increase with the increase in the h-BN content. This is due to the higher rigidity of h-BN compared to the matrix resin, and its incorporation enhances the mechanical stability of the material [37,38,39]. Furthermore, h-BN penetrates the amorphous part of the polymer, thereby enhancing the rigidity and strength of the polymer material. Simultaneously, the incorporation of h-BN promotes heterogeneous nucleation in the polymer, further improving the mechanical stability of the material [40,41]. These factors collectively lead to a gradual increase in the storage modulus of the PA5T-CO-10T/BN composite material as the h-BN content increases.

As depicted in Figure 6b, the loss modulus of the PA5T-CO-10T/BN composites with different h-BN contents gradually decreases with increasing temperatures. This is due to the fact that as the polymer gradually melts, the movement of the polymer molecular chains becomes more free, and the internal friction between the molecular chains decreases, resulting in a significant decrease in the loss modulus with increasing temperatures [42]. From Figure 6b, it can also be observed that the loss modulus of the material gradually increases with the increase in the h-BN content. This is due to the silane coupling agent binding the matrix resin and h-BN together, thereby enhancing the intermolecular adhesion [43]. This creates obstacles during the movement of molecular chains, leading to an increase in energy loss during the flow of the melt, thus gradually increasing the loss modulus. Additionally, the storage modulus of PA5T-CO-10T is lower than its loss modulus. However, when the h-BN content reaches 30%, the storage modulus of PA5T-CO-10T/30 wt%BN is higher than its loss modulus, indicating that as the h-BN content increases, the material transitions from having viscous to elastic characteristics.

As indicated in Figure 6c, similar to the trends of the storage modulus and loss modulus, the complex viscosity of the PA5T-CO-10T/BN composites with varying h-BN contents gradually decreases as the temperature rises. Meanwhile, as the amount of h-BN added increases, the complex viscosity gradually increases. This is due to the intensification of the polymer molecular chain segment motion as the temperature rises, which leads to an increase in the intermolecular distance and a decrease in the intermolecular forces, consequently resulting in a reduction in the complex viscosity. Furthermore, as the content of h-BN increases, the complex viscosity of the material gradually rises. This is attributed to the strong bonding between the matrix resin and h-BN, as the incorporation of h-BN hinders the motion of the polymer molecular chains [44]. The incorporation of h-BN significantly enhances the thermal conductivity of polymers, but it also results in a decline in the material’s processing fluidity. Therefore, when determining the amount of h-BN to add, especially when processing thin-walled products, it is imperative to thoroughly consider the influence of h-BN on both the thermal conductivity and viscosity of the polymer and to select an appropriate addition level accordingly.

### 3.7. XRD Analysis

**Figure 7:**
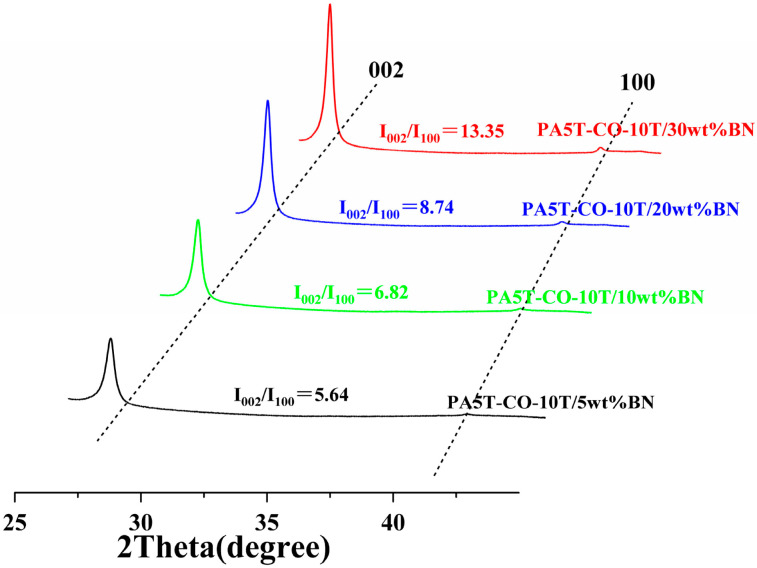
X-ray diffraction orientation analysis of PA5T-CO-10T/BN composite with different h-BN contents.

Figure 7 presents the X-ray diffraction spectra of the PA5T-CO-10T/BN composites with different h-BN contents. The diffraction peaks at 27.6° and 41.6° correspond to the (002) and (100) plane diffraction peaks of h-BN, respectively. The diffraction peak in the horizontal direction is represented by I002, and the diffraction peak in the vertical direction is represented by I100. The I002/I100 ratio is commonly used to indicate the degree of orientation. As depicted in Figure 7, when the content of h-BN ranges from 5 to 30 wt%, the I002/I100 ratio falls between 5.64 and 13.35. Most of the h-BN exhibits a high level of in-plane orientation, potentially due to the flow of the injected melt during processing. Additionally, Figure 7 reveals that as the h-BN content increases, the horizontal orientation of h-BN in the PA5T-CO-10T/BN composite gradually increases. This is attributed to the reduced degree of freedom in motion during shear flow with the increasing amount of h-BN added, leading to a more pronounced orientation structure of h-BN in the resulting composite, which also provides a guarantee for the continuous improvement of the material’s thermal conductivity [44,45]. This is consistent with the SEM analysis results presented in Section 3.3.

## 4. Conclusions

In this paper, we prepared bio-based PA5T-CO-10T/BN composites by blending as-synthesized PA5T-CO-10T with h-BN. The microstructure, thermal conductivity, storage modulus, loss modulus, complex viscosity and crystallization properties of the PA5T-CO-10T/BN composites with varying h-BN contents were investigated. The results indicate that as the content of h-BN increases, the thermal conductivity of the PA5T-CO-10T/BN composite gradually increases. When the mass of h-BN reaches 30 wt%, the thermal conductivity of the composite is 2.5 times that of the original matrix resin. Simultaneously, the storage modulus, loss modulus, complex viscosity and h-BN orientation degree of the material all exhibit a significant upward trend with the increase in the h-BN content. This is attributed to the high thermal conductivity and the high degree of orientation of h-BN, which provide a guarantee for the continuous enhancement of the material’s thermal conductivity. Additionally, the incorporation of h-BN fills the amorphous parts of the polymer, facilitates heterogeneous nucleation and enhances the interconnections between molecular chains. The PA5T-CO-10T/BN composites exhibit excellent processability and remarkable heat resistance and thermal conductivity, making them suitable as structural materials for applications in electronics, electrical appliances and automobiles.

## Figures and Tables

**Table 1:** Raw material ratios of PA5T-CO-10T and PA5T-CO-10T/BN.

Samples	PA5T-CO-10T wt%	h-BN wt%	Antioxidant 1010 wt%
PA5T-CO-10T	97	0	3
PA5T-CO-10T/5 wt%BN	92	5	3
PA5T-CO-10T/10 wt%BN	87	10	3
PA5T-CO-10T/20 wt%BN	77	20	3
PA5T-CO-10T/30 wt%BN	67	30	3

## Data Availability

The data that support the findings of this study are available on request from the corresponding author. The data are not publicly available due to privacy or ethical restrictions.

## References

[1] Zhang G., Yan G.-M., Yu T., Lu J.-H., Huang X., Wang X.-J., Yang J. (2017). Semiaromatic Polyamides Containing Carboxyl Unit: Synthesis and Properties. Ind. Eng. Chem. Res..

[2] Yan G.-M., Wang H., Li D.-S., Lu H.-R., Liu S.-L., Yang J., Zhang G. (2021). Design of recyclable, fast-responsive and high temperature shape memory semi-aromatic polyamide. Polymer.

[3] Ai T., Feng W., Zou G., Ren Z., Wang P., Ji J., Zhang W. (2020). High-performances biobased semi-aromatic polyamide10Tcopolymerized with silicone monomers. J. Appl. Polym. Sci..

[4] Feng W., Zou G., Ding Y., Ai T., Wang P., Ren Z., Ji J. (2019). Effect of Aliphatic Diacid Chain Length on Properties of Semiaromatic Copolyamides Based on PA10T and Their Theoretical Study. Ind. Eng. Chem. Res..

[5] Yang S.H., Fu P., Liu M.Y., Wang Y.D., Zhang Y.C., Zhao Q.X. (2010). Synthesis, characterization of polytridecamethylene 2,6-naphthalamide as semiaromatic polyamide containing naphthalene-ring. Express Polym. Lett..

[6] Feng J. (2020). Failure Analysis of Rheocast Cylinder Head Covers of Hypereutectic Al–Si Alloys. J. Fail. Anal. Prev..

[7] Yang H., Yang K., Mai J., Jiang S., Jiang Z. (2019). Synthesis and Application of Semi-aromatic Heat-resistant Nylon PA6T/10T. Engi-Neering Plast. Appl..

[8] Altintas O., Romaire J.P., Perkins D.L., Sun T., Wang L., Patel N., Callen N.M., Burns A.B., Gopinadhan M. (2023). Molecular-Level Control of Thermal and Morphological Transitions in Semi-Aromatic Polyamides by Cu(I)-Catalyzed Azide–Alkyne Click Polymerization. Macromolecules.

[9] Shen T., Zhang B., Wang Y., Yang P., Li M., Hu R., Guo K., Chen K., Zhu N., Wang L. (2022). Production of 100% bio-based semi-aromatic nylon by aerobic oxidation of 5-hydroxymethylfurfural to 2,5-furandicarboxylic acid with bio aliphatic diamine. Chem. Eng. J..

[10] Xie S., Yang J., Wang X., Yang J. (2022). Synthesis of fully biobased semi-aromatic furan polyamides with high performance through facile green synthesis process. Eur. Polym. J..

[11] Zhang G., Yan G.-M., Ren H.-H., Li Y., Wang X.-J., Yang J. (2016). Effects of a trans- or cis-cyclohexane unit on the thermal and rheological properties of semi-aromatic polyamides. Polym. Chem..

[12] Chapman S.K., Glant S.K. (2010). Antiproliferative effects of inhibitors of polyamine synthesis in tumors of neural origin. J. Pharm. Sci..

[13] Cao J.-P., Zhao J., Zhao X., You F., Yu H., Hu G.-H., Dang Z.-M. (2013). High thermal conductivity and high electrical resistivity of poly(vinylidene fluoride)/polystyrene blends by controlling the localization of hybrid fillers. Compos. Sci. Technol..

[14] Yu R.-L., Zhang L.-S., Feng Y.-H., Zhang R.-Y., Zhu J. (2014). Improvement in toughness of polylactide by melt blending with bio-based poly(ester)urethane. Chin. J. Polym. Sci..

[15] Miao X., Wang C., Liao T., Ju S., Zha J., Wang W., Liu J., Zhang Y., Ren Q., Xu F. (2023). Novel magnetocaloric composites with outstanding thermal conductivity and mechanical properties boosted by continuous Cu network. Acta Mater..

[16] Liu L., Feng Y.P., Shen Z.X. (2003). Structural and electronic properties of *h*-BN. Phys. Rev. B.

[17] Chen C., Guo Z., Ji L., Gao H., Hao J., Ye A. (2016). Fabrication of Al-Cu Composite Reinforced with BN by Powder Liquid-Phase Forging. Rare Met. Mater. Eng..

[18] Wang Z., Zhang K., Zhang B., Tong Z., Mao S., Bai H., Lu Y. (2022). Ultrafast battery heat dissipation enabled by highly ordered and interconnected hexagonal boron nitride thermal conductive composites. Green Energy Environ..

[19] Zhao C.B., Xu S.C., Qin Y.F., Su L., Yang X.J. (2014). Thermal Conductivity Cyanate Ester Resin Composites Filled with Boron Nitride. Adv. Mater. Res..

[20] Zhang Y.M., Wang H.B., He X.D., Han J.C., Du S.Y. (2001). Fabrication of hexagonal boron nitride based ceramics by combustion synthesis. Trans. Nonferrous Met. Soc. China.

[21] Ji Y., Han S.-D., Wu H., Guo S.-Y., Zhang F.-S., Qiu J.-H. (2023). Understanding the Thermal Impedance of Silicone Rubber/Hexagonal Boron Nitride Composites as Thermal Interface Materials. Chin. J. Polym. Sci..

[22] Chen H., Wang Y., Nan Y., Wang X., Yue X., Zhang Y., Fan H. (2024). Effects of BN on the Mechanical and Thermal Properties of PP/BN Composites. J. Wuhan Univ. Technol. Sci. Ed..

[23] Liu B., Zhang S., Ma L., Wu Y., Li C., Wu Z., Bian X., Yan W. (2023). Synthesis, characterization and crystallization kinetics of a bio-based, heat-resistance nylon 5T/10T. RSC Adv..

[24] (2024). Plastics-Determination of Thermal Conductivity and Thermal Diffusivity.

[25] Kint D.P.R., Muñoz-Guerra S. (2003). Modification of the thermal properties and crystallization behaviour of poly(ethylene terephthalate) by copolymerization. Polym. Int..

[26] Meng C., Liu X. (2022). Study on reaction kinetics of bio-based semi-aromatic high-temperature polyamide PA5T/56. Polym. Bull..

[27] Andrews L., Wang X. (2002). Infrared Spectrum of the Novel Electron-Deficient BH_4_ Radical in Solid Neon. J. Am. Chem. Soc..

[28] Luo F.-H., Dong Z.-T., Chen G.-H., Ma C., Wang H.-Y. (2024). Preparation of PVA/GO/h-BN Janus Film with High Thermal Conductivity and Excellent Flexibility via a Density Deposition Self-assembly Method. Chin. J. Polym. Sci..

[29] Gu J., Zhang Q., Dang J., Xie C. (2012). Thermal conductivity epoxy resin composites filled with boron nitride. Polym. Adv. Technol..

[30] Liang Q., Xiu Y., Lin W., Moon K.-S., Wong C.P. Epoxy/h-BN composites for thermally conductive underfill material. Proceedings of the 2009 IEEE 59th Electronic Components and Technology Conference (ECTC 2009).

[31] Zhang Z.-W., Zheng W.-T., Jiang Q. (2012). Hydrogen adsorption on Ce/BNNT systems: A DFT study. Int. J. Hydrogen Energy.

[32] Cao M., Zhang C., He B., Huang M., Jiang S. (2017). Synthesis of 2,5-furandicarboxylic acid-based heat-resistant polyamides under existing industrialization process. Macromol. Res..

[33] Tiwari R., Butola B.S., Joshi M. (2025). Effect of Ethoxy functionalized hexagonal boron nitride on h-BN/TPU thermoplastic polyurethane nanocomposite and its thermal properties. Polym. Eng. Sci..

[34] Ma T., Zhao Y., Ruan K., Liu X., Zhang J., Guo Y., Yang X., Kong J., Gu J. (2019). Highly Thermal Conductivities, Excellent Mechanical Robustness and Flexibility, and Outstanding Thermal Stabilities of Aramid Nanofiber Composite Papers with Nacre-Mimetic Layered Structures. ACS Appl. Mater. Interfaces.

[35] Ng H.Y., Lu X., Lau S.K. (2010). Thermal conductivity of boron nitride-filled thermoplastics: Effect of filler characteristics and composite processing conditions. Polym. Compos..

[36] Zhang Y., Peng Y., Zhang X., Fu X., Liu Z. (2017). Influence of Coupling Agents on Thermally Conductive Properties of Polyamide 6/Boron Nitride Composites and Optimization for Their Preparation Technology. China Plast..

[37] Zhang Z., Duan X., Qiu B., Chen L., Zhang P., Cai D., He P., Zhang H., Wei Z., Yang Z. (2020). Microstructure evolution and grain growth mechanisms of h-BN ceramics during hot-pressing. J. Eur. Ceram. Soc..

[38] Chen H., Xiao G., Chen Z., Yi M., Zhang J., Li Z., Xu C. (2022). Hexagonal boron nitride (h-BN) nanosheets as lubricant additive to 5CB liquid crystal for friction and wear reduction. Mater. Lett..

[39] Huang X., Zhang J., Cheng Y., Chen C., Lian G., Jiang J., Feng M., Zhou M. (2020). Effect of h-BN addition on the microstructure characteristics, residual stress and tribological behavior of WC-reinforced Ni-based composite coatings. Surf. Coat. Technol..

[40] Zhang D., Wu F., Ying Q., Gao X., Li N., Wang K., Yin Z., Cheng Y., Meng G. (2019). Thickness-tunable growth of ultra-large, continuous and high-dielectric h-BN thin films. J. Mater. Chem. C.

[41] Chen B., Bi Q., Yang J., Xia Y., Hao J. (2008). Tribological properties of solid lubricants (graphite, h-BN) for Cu-based P/M friction composites. Tribol. Int..

[42] Jiang L., Zhang X., Zhou Y.-H. (2023). Nonlinear static and dynamic mechanical behaviors of Nb3Sn superconducting composite wire: Experiment and analysis. Acta Mech. Sin..

[43] Tyagi R., Xiong D.S., Li J.L., Dai J. (2010). High-Temperature Friction and Wear of Ag/h-BN-Containing Ni-based Composites Against Steel. Tribol. Lett..

[44] Kostoglou N., Polychronopoulou K., Rebholz C. (2015). Thermal and chemical stability of hexagonal boron nitride (h-BN) nanoplatelets. Vacuum.

[45] Gyawali G., Adhikari R., Kim H.S., Cho H.-B., Lee S.W. (2012). Effect of h-BN Nanosheets Codeposition on Electrochemical Corrosion Behavior of Electrodeposited Nickel Composite Coatings. ECS Electrochem. Lett..

